# Beyond the Limits of Oxygen: Effects of Hypoxia in a Hormone-Independent Prostate Cancer Cell Line

**DOI:** 10.1155/2013/918207

**Published:** 2013-09-12

**Authors:** A. C. Mamede, A. M. Abrantes, L. Pedrosa, J. E. Casalta-Lopes, A. S. Pires, R. J. Teixo, A. C. Gonçalves, A. B. Sarmento-Ribeiro, C. J. Maia, M. F. Botelho

**Affiliations:** ^1^Biophysics Unit, Institute of Biomedical Research on Light and Image (IBILI), Faculty of Medicine, University of Coimbra, Azinhaga de Santa Comba, Celas, 3000-548 Coimbra, Portugal; ^2^CICS-UBI, Health Sciences Research Centre, University of Beira Interior, Covilhã, Portugal; ^3^Centre of Investigation on Environment, Genetics and Oncobiology (CIMAGO), Faculty of Medicine, University of Coimbra, Coimbra, Portugal; ^4^Faculty of Sciences and Technology, University of Coimbra, Coimbra, Portugal; ^5^Applied Molecular Biology and Hematology Group, Faculty of Medicine, University of Coimbra, Coimbra, Portugal

## Abstract

Prostate cancer (PCa) has a high incidence worldwide. One of the major causes of PCa resistance is intratumoral hypoxia. In solid tumors, hypoxia is strongly associated with malignant progression and resistance to therapy, which is an indicator of poor prognosis. The antiproliferative effect and induced death caused by doxorubicin, epirubicin, cisplatin, and flutamide in a hormone-independent PCa cell line will be evaluated. The hypoxia effect on drug resistance to these drugs, as well as cell proliferation and migration, will be also analyzed. All drugs induced an antiproliferative effect and also cell death in the cell line under study. Hypoxia made the cells more resistant to all drugs. Moreover, our results reveal that long time cell exposure to hypoxia decreases cellular proliferation and migration. Hypoxia can influence cellular resistance, proliferation, and migration. This study shows that hypoxia may be a key factor in the regulation of PCa.

## 1. Introduction

Prostate cancer (PCa) is one of the most common diseases in the world, being the sixth leading cause of cancer death worldwide [1]. The incidence of PCa varies according to three major risk factors: age, ethnicity, and familial predisposition [2–6]. 

Hypoxia can be defined as a reduction in oxygen partial pressure (pO_2_). This characteristic constitutes a condition of various pathophysiological states such as ischemic vascular disease, myocardial infarction, stroke, respiratory insufficiency, and cancer [7]. Clinically relevant levels of hypoxia are detected in 50% to 60% of solid tumors [8]. Hypoxia results from an imbalance between rate of consumption and supply of oxygen to cancer cells, thus compromising several cellular functions. Cancer cells have the capacity to adapt to hypoxic environments due to various genetic and epigenetic mechanisms and alterations at cellular level that contribute to adaptive changes which lead to a clinically aggressive phenotype, having hypoxic tumors a poor prognosis. These tumors are more difficult to treat, being highly resistant, presenting an increased risk of recurrence and progression [9, 10]. 

Regarding PCa, intratumoral oxygen levels were evaluated through a polarographic electrode with the form of a needle, and it was found that pO_2_ in PCa was significantly lower (pO_2_ = 2.4 mmHg) than in normal tissues (muscle, pO_2_ = 30 mmHg) [11]. In fact, increase of hypoxia may be critical in PCa progression and resistance [12]. 

In this work, we aim at studying the antiproliferative effect and cell death induced by various agents of chemotherapy and hormonal therapy in a human hormone-independent PCa cell line. Response to these treatment agents will be evaluated in normoxia and hypoxia conditions. Chemotherapy is a treatment used in PCa when it stops responding to hormone therapy. However, several studies show that some antiandrogens can have an inhibitory effect, independent of androgen receptor expression, in advanced PCa. Cellular proliferation and migration of the cell line under study will be determined under normoxia and hypoxia environments.

## 2. Methods

### 2.1. Cell Culture

Human hormone-independent PCa cell line (PC3), acquired from American Type Culture Collection (Rockville, MD, USA), was cultured in RPMI-1640 (Sigma R4130) supplemented with 100 *μ*M sodium pyruvate (Gibco 11360), 10% fetal bovine serum (Sigma F7524), and 1% antibiotic/antimycotic (Gibco 15140-122). Cells were maintained at 37°C and 5% CO_2_ in a cell incubator (Heraeus HeraCell 150 CO_2_ Incubator—BridgePath Scientific, MD, USA).

### 2.2. Evaluation of Antiproliferative Effect Induced by Chemotherapy and Hormonal Therapy

Chemotherapy agents (doxorubicin, epirubicin, and cisplatin), as well as an antiandrogen (flutamide), were used. In this study, cells were incubated with different concentrations of these drugs. After 24, 48, 72, and 96 h of drug stimulation, cell proliferation was evaluated by the colorimetric test MTT (3-(4,5-dimethylthiazol-2)2,5-diphenyltetrazolium bromide), as previously described by Mamede et al. [13]. The obtained results were analyzed and processed in software OriginPro 8.0, being the cytotoxicity expressed as a percentage of inhibition of cell proliferation when compared to control experiments. This allows the determination of the concentration that inhibits cell proliferation in 50% (IC_50_) through a sigmoid fitting (Boltzmann function). 

### 2.3. Characterization of Cell Death Induced by Chemotherapy and Hormonal Therapy

Cells were incubated with three concentrations of the drugs for 48 h (Table 1). The first concentration (*C*1) corresponds to a drug concentration that does not inhibit cell proliferation, the second concentration (*C*2) corresponds to IC_50_ and, finally, the third concentration (*C*3) corresponds to a concentration that totally inhibits cell proliferation (values based on dose-response curves obtained for each drug after 48 h of incubation). Annexin-V/propidium iodide (AV/PI) incorporation assay was used, as already described by Abrantes et al. [14].

### 2.4. Normoxia versus Hypoxia

The following studies were performed in normoxia (95% O_2_ and 5% CO_2_), and in hypoxia (93% N_2_, 2% O_2_, and 5% CO_2_) conditions. Studies in hypoxia were performed in a controlled environment chamber (Plas-Labs, Lansing, MI).

### 2.5. Evaluation of Cellular Resistance to Chemotherapy and Hormonal Therapy in Hypoxia

Cells were incubated with IC_50_ determined in normoxia for each drug. These experiments were performed during 48 h in hypoxia and normoxia conditions. After 48 h, cells were submitted to MTT proliferation assay.

### 2.6. Evaluation of Cell Proliferation in Hypoxia

A cell suspension of 70000 cells/mL was distributed in 12-well-plates. After 24 h, cells were placed in hypoxia and normoxia. The cells in normoxia conditions were considered as controls. After 48 h, mitochondrial activity was assessed by MTT assay, according to the procedure described above, and protein synthesis was evaluated through the sulforhodamine B (SRB) assay according to Houghton et al. [15]. SRB is an anionic purple dye that binds to proteins through electrostatic bonds. The fixed dye can be measured by spectrophotometry after solubilization, which correlates with the rate of total protein synthesis. The contents of each well were transferred to a plate with 96 wells, and the absorbance was quantified at 540 nm with a reference filter of 690 nm in an ELISA spectrophotometer (SLT Spectra).

### 2.7. Evaluation of Cell Migration in Hypoxia

Scratch assay is commonly used to evaluate cell migration *in vitro*, according to Liang et al. [16]. In this study, cells were plated in Petri dishes. When the cellular confluence reached 90%, a risk was drawn using a tip in order to separate the monolayer. Immediately after (0 h) and at regular intervals of time (8 and 24 h), images were captured (100x). Cells were photographed with a microscope Motic AE31 through the system Motic Images Advanced 3.2. Images were compared in order to quantify the rate of closure by (1), where *A*_0_ is the initial area of risk (when *t* = 0 h), *A*_*t*_ corresponds to risk area at time *t* (when *t* = 8 h or *t* = 24 h), and *T* is time in hours. This test was conducted in hypoxia and normoxia conditions, and rate of risk closure in both conditions and its standard deviation were calculated

(1) $$\begin{array}{c} \text{rate}\;\text{of}\;\text{closure}\;\left(\%\right)=\frac{A_{0}-A_{t\;}}{T}.\;\; \end{array}$$

### 2.8. Statistical Analysis

Statistical analysis was performed using IBM SPSS v.19 software. In the descriptive analysis, measures of central tendency (mean and median) and dispersion (standard deviation and interquartile range) for quantitative variables were determined. The normal distribution of these variables was assessed using the Shapiro-Wilk test. In the case of normal underlying distributions, parametric tests were used to make comparisons, and in the opposite case we used nonparametric tests. The comparison of quantitative variables between two groups was performed using Student *t* test (parametric) and Mann-Whitney test (nonparametric). Comparison of quantitative variables in more than two groups was obtained using one-factor ANOVA test with post hoc analysis using the Tuckey test (parametric tests) and the Kruskal-Wallis test, with multiple comparisons performed using the Mann-Whitney test with Bonferroni correction. It was considered a significance level of 5%.

## 3. Results

**Figure 1:**
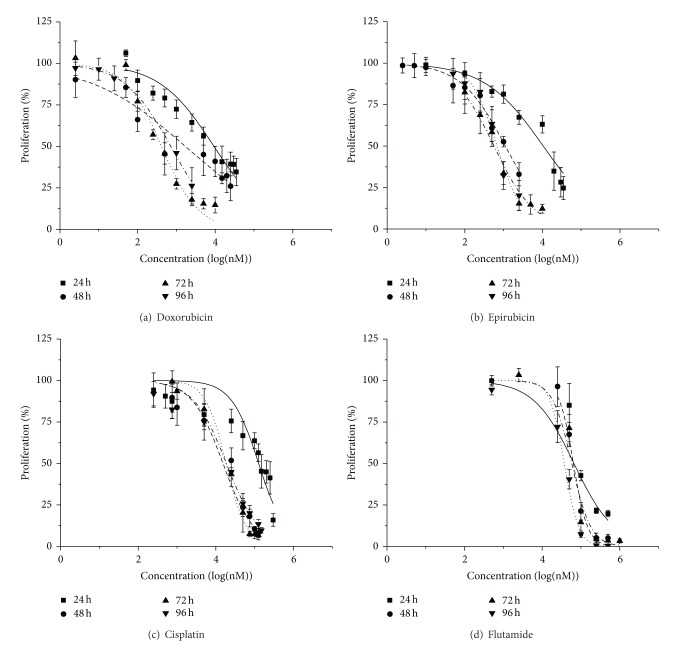
Dose-response curves resulting from incubation of PC3 cell line with doxorubicin (a), epirubicin (b), cisplatin (c), and flutamide (d). Each experiment was performed in duplicate and repeated in three different sets of tests.

Pharmacological response of PC3 cell line to doxorubicin, epirubicin, cisplatin, and flutamide for incubation times of 24, 48, 72, and 96 h were shown at Figures 1(a), 1(b), 1(c), and 1(d). 

Cell proliferation decreases with increasing drug concentration and incubation period, except for doxorubicin, epirubicin, and cisplatin at 96 h, in which there is a significant increase (*P* < 0.05) compared to 72 h (Table 2). Incubation with doxorubicin and epirubicin resulted in similar IC_50_ values over time. It is worth noting that the proliferation was more easily inhibited with doxorubicin and epirubicin than with cisplatin or flutamide.

**Figure 2:**
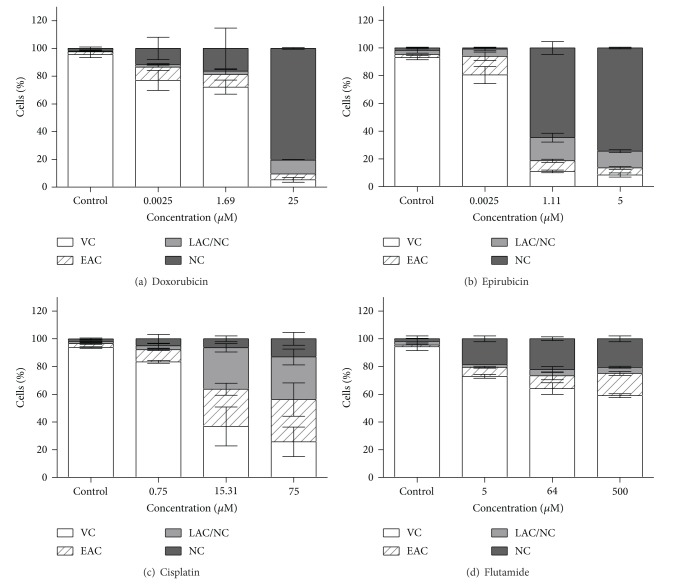
Cell viability analyzed by flow cytometry using double staining with AV/PI. Schematic representation of the percentage of viable cells (VC), in initial apoptosis (EAC), in late apoptosis/necrosis (LAC/NC), and necrosis (NC) after incubation with doxorubicin (a), epirubicin (b), cisplatin (c), and flutamide (d). Data express the mean and standard deviation of three independent experiments.

After cell proliferation, the identification of cell death was performed by flow cytometry using the double staining AV/PI. The results showed that as the concentration of drugs increases, the number of viable cells decreases in a dose-dependent manner, as shown in Figure 2. Cell death increases in response to increasing doxorubicin concentration. Cell death occurred at all concentrations predominantly by necrosis, as observed in Figure 2(a). By IC_50_ (1.69 03BCM) the cell death was 27%. Regarding the results obtained after cell incubation with epirubicin, it was observed that cell death also increases in response to increase of drug concentration (Figure 2(b)). At the lowest epirubicin concentration the cell death occurred predominantly by initial apoptosis (14%), which was statistically significant when compared to control (*P* < 0.05). However, at the highest concentration used (5 *μ*M), cell death occurred mainly through necrosis. With the IC_50_ (1.11 *μ*M), the viability only reached 11%.

The cell death increased by increasing cisplatin concentration (Figure 2(c)). At IC_50_ (15.31 *μ*M), cell death occurred predominantly by initial (27%) and late (30%) apoptosis. Like the IC_50_, also with the higher concentration (75 *μ*M) there is a predominance of cell death by initial (30%) and late (31%) apoptosis. These results were significantly different from control (*P* < 0.05). Cell viability decreases by increasing flutamide concentration, as shown in Figure 2(d) (no significant differences between concentrations were obtained). The cell death induced by flutamide did not exceed 50%.

**Figure 3:**
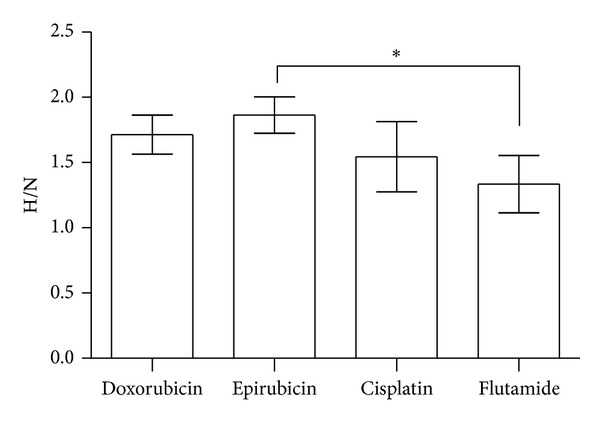
Cellular resistance to treatment with chemotherapy (cisplatin, doxorubicin, and epirubicin) and hormonal therapy (flutamide). The results are expressed as the ratio between the calculated proliferation in hypoxia and normoxia and represent the mean and standard deviation of six independent experiments.

A ratio between proliferation in hypoxia and normoxia conditions was calculated in order to analyze cell drug resistance in hypoxia (Figure 3). Flutamide had the lowest ratio between hypoxia/normoxia (H/N = 1.34), whereas epirubicin had a significantly higher ratio H/N = 1.87 (*P* = 0.032).

**Figure 4:**
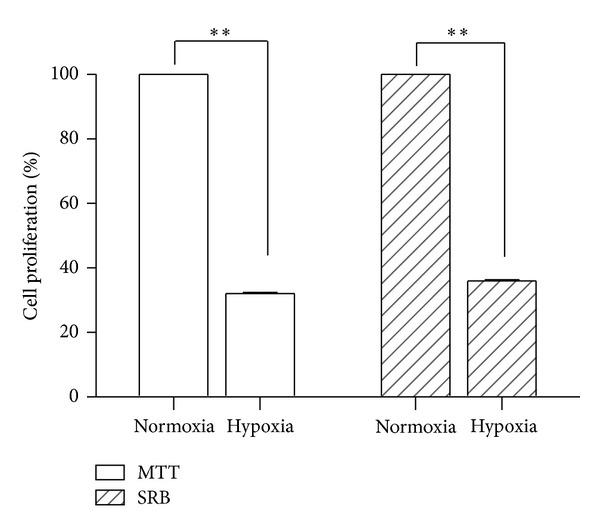
Cell proliferation in hypoxia, determined by MTT and SRB assay, relatives to normoxia and its standard deviation. The results represent the mean and standard deviation of six independent experiments.

The results concerning the analysis of cell proliferation in hypoxia and normoxia through MTT and SRB assays showed that there is a decrease in cell proliferation in hypoxia. As shown in Figure 4, the results obtained by MTT and SRB assays are similar. However, in hypoxia there was a decrease of 68% and 64% of cell proliferation through MTT and SRB, respectively.

**Figure 5:**
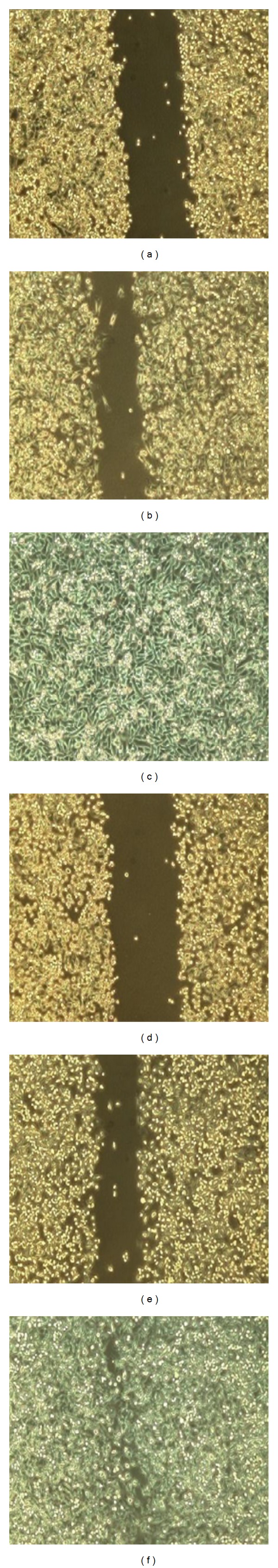
Cell migration at 0 ((a) and (d)), 8 ((b) and (e)), and 24 hours ((c) and (f)) in normoxia ((a), (b), and (c)) and hypoxia ((d), (e), and (f)). Cells were photographed through an optical microscope at a magnification of 100x.

Scratch assay revealed that there was a decrease in the risk area over time, as observed in Figure 5. After 8 h, we still observed no differences in migration between the two conditions, being however a visible decreased risk area as compared to 0 h in the same environment. Nevertheless, after 24 h, the risk in normoxia is completely closed (Figure 5(c)), but not in hypoxia condition (Figure 5(f)). 

Regarding the closure risk rate, there was a decrease in this value over time, being higher in normoxia than in hypoxia (Table 3). 

## 4. Discussion 

With hypoxia being strongly associated with malignant progression and resistance, this condition becomes a central issue in cancer treatment [12]. For this reason, it is important to study the effect of hypoxia in PCa, particularly learning about the influence of this microenvironment in cell resistance, proliferation, and migration.

In PCa treatment, hormonal therapy has particular importance. When PCa cells are androgen dependent, it is important to use therapeutic strategies that are based on inhibition of androgen receptor (AR) using antagonists of AR, such as flutamide [17]. When PCa cells become hormone resistant, hormonal therapy becomes ineffective, and it is necessary to use, for example, conventional chemotherapy. Taking into account that this form of treatment is largely palliative and not curative, chemotherapy is still subject of study in this type of cancer, being not used as the first choice in PCa treatment [18].

Through MTT results, it can be concluded that there was a decrease in the IC_50_ values over time with all drugs, However, there was a significant increase (*P* < 0.05) in IC_50_ value at 96 h compared to 72 h after cell incubation with doxorubicin, epirubicin, or cisplatin. This may be due to the acquisition of cellular chemoresistance over time. Cells are more sensitive to doxorubicin and epirubicin than cisplatin. Smith et al. [19] conducted studies in another PCa cell line (LNCaP), which is AR positive, and concluded that cisplatin treatment enhances the expression of metallothioneins, molecules that induce drug resistance. Metallothioneins are a family of proteins which play a role in resistance to chemotherapy and radiotherapy. Their expression is upregulated in response to presence of metal ions, such as zinc, which is highly concentrated in prostatic tissue [19]. The resistance to cisplatin may be related to the expression of the protein zinc-finger protein 143 (ZNF143), which is induced by treatment with agents that damage DNA. Wakasugi et al. [20] studied the PC3 resistance to cisplatin and demonstrated that ZNF143 protein is overexpressed in cells resistant to cisplatin, since it found that inhibition of ZNF143 increased the cisplatin sensitivity of PC3. Although PC3 does not express AR, it has been demonstrated that flutamide inhibits proliferation of AR negative PCa cells [21–23]. However, this mechanism of inhibition remains to be elucidated.

Through flow cytometry studies, it was observed that doxorubicin IC_50_ incubation results in a 73% of cell viability, while epirubicin incubation results in a much lower viability. In addition, our results suggest that doxorubicin only inhibits cellular proliferation, while epirubicin inhibits cellular proliferation and causes cell death. Regarding the cell treatment with flutamide, cell death did not exceed 50%, indicating that flutamide inhibits cell proliferation but does not induce cell death.

All assays in hypoxia were performed over a long period of time (48 h), to better mimic the real tumor environment where tumor vascularization is insufficient and oxygen diffusion into the tissues is limited. It is well known that the development of PCa is not only associated with increasing age but also with increased hypoxia [24].

Our results show a great cellular resistance when cells are incubated with epirubicin in hypoxic conditions but not with cisplatin incubation. Flutamide has a lower H/N ratio than all drugs used. Several studies corroborate the results obtained in this work. Frederiksen et al. [25] showed that PCa cells are resistant to doxorubicin in hypoxia probably due to mutation or inactivation of P53 and overexpression of Bcl-2. Other studies have demonstrated that Bcl-xL, an antiapoptotic protein of the Bcl-2 family, is overexpressed in PCa [26]. Frederiksen et al. [25] also claim that resistance to anticancer drugs can be attributed in part to the increase of multidrug resistance transporters, including P-glycoprotein (PgP). In fact, PgP is upregulated in PC3 cells in response to extracellular acidosis induced by hypoxia due to accumulation of high lactate and low glucose concentration associated with anaerobic glycolysis [27]. 

 To investigate the influence of hypoxia on cell proliferation, MTT and SRB methods were used, which assess the viability of the mitochondria and the rate of protein synthesis, respectively. The results showed that cell proliferation decreases in long hypoxia exposure, as confirmed by both methodologies.

Scratch assay is based on the observation of cells in confluent monolayer, that, being on the edge of the slot created, moves to be close until cell-cell contacts are established again. The results of cell migration can be correlated with cell proliferation: in hypoxic environment there is a lower cell migration, since after 24 h exposure to hypoxia, cells showed no ability to migrate thereby “closing” the risk completely, unlike what happened in normoxia. Thus, there was a decrease not only in proliferation but also in cell migration. Regarding the closure rate risk, there are a decrease over time and also a decrease in hypoxia. Dai et al. [28] assessed PC3 migration during prolonged (>24 h) or short time (<6 h) exposure to hypoxia and concluded that less exposure to hypoxia results in a significant increase in cell migration, while longer exposure inhibited cell migration, a result that corroborates the results obtained in this study. For more than 24 h exposure to hypoxia, these authors found an increase in expression of genes involved in tumor invasion, including matrix metalloproteinase 2 (MMP-2) [29]. Studies performed *in vivo* with lung cancer cells show that prolonged exposure to hypoxia inhibits tumor progression in mice. Controversially, the same study concluded that, under the same conditions, tumor growth of human colorectal cancer was increased [30]. So, the authors concluded that mechanisms for response to hypoxia depend on the cell type and exposure period.

Through this study it can be concluded that hypoxia is a key factor in the regulation of PCa. Although this subject is being increasingly studied, it is important to know more about the influence of hypoxia in PCa, as well as on other cancers, on order to develop better diagnosis and treatment techniques.

## Figures and Tables

**Table 1:** Drugs concentration (*μ*M) for flow cytometry studies.

	*C*1 (*μ*M)	*C*2 (μM)	*C*3 (*μ*M)
Doxorubicin	0.0025	1.69	25
Epirubicin	0.025	1.11	5
Cisplatin	0.75	15.31	75
Flutamide	5	64	500

**Table 2:** IC_50_ (*μ*M) obtained for PC3 cell line after incubation with doxorubicin, epirubicin, cisplatin, and flutamide at 24, 48, 72, and 96 hours of exposure. *R* ^2^ is also indicated.

Drug	Time (h)	IC_50_ (*μ*M)	*R* ^2^
Doxorubicin	24 48 72 96	9.31 1.69 0.42 0.71	0.88 0.94 0.95 0.99
Epirubicin	24 48 72 96	10.55 1.11 0.57 0.68	0.95 0.99 0.92 0.97
Cisplatin	24 48 72 96	127.08 15.31 18.35 18.76	0.83 0.98 0.99 0.97
Flutamide	24 48 72 96	66.25 64.00 57.99 37.36	0.97 0.95 0.97 0.98

**Table 3:** Rate of closure risk in normoxia and hypoxia and its standard deviation. The results represent the average of three independent experiments.

		Normoxia (MED ± SD)	Hypoxia (MED ± SD)
Time (h)	8	5.65 ± 2.30	2.1 ± 1.45
	24	3.51 ± 2.34	1.5 ± 0.93
